# A Metatranscriptomic Approach to the Identification of Microbiota Associated with the Ant *Formica exsecta*

**DOI:** 10.1371/journal.pone.0079777

**Published:** 2013-11-18

**Authors:** Helena Johansson, Kishor Dhaygude, Stafva Lindström, Heikki Helanterä, Liselotte Sundström, Kalevi Trontti

**Affiliations:** Centre of Excellence in Biological Interactions, Department of Biosciences, University of Helsinki, Helsinki, Finland; Sheffield University, United States of America

## Abstract

Social insects live in cooperative colonies, often in high densities and with closely related individuals, and interact using social contact behaviours. Compared to solitary insects, social insects have evolved multi-level immunity that includes immune responses common to holometabolous insects, and social immunity, which is exclusive to social taxa. This suggests that social insects may be subject to high pathogen pressure, yet relatively little is known about the range of symbiotic and pathogenic microbial communities that associate with social insects. In this study we examined transcriptome data generated from the ant *Formica exsecta* for sequences identifying as microbes (or other organisms potentially of non-ant origin). Sequences showing homology to two viruses and several other potentially or obligate intracellular organisms, such as *Wolbachia, Arsenophonus, Entomoplasmatales* and *Microsporidia,* were present in the transcriptome data. These homologous sequence matches correspond to genera/species that have previously been associated with a variety of insects, including social insects. There were also sequences with identity to several other microbes such as common moulds and soil bacteria. We conclude that this sequence data provides a starting point for a deeper understanding of the biological interactions between a species of ant and the micro- and macrobiotic communities that it potentially encounters.

## Introduction

Identifying and classifying pathogens for a species of interest has great significance for fundamental research in ecology and evolution. The recent advent of genomic and transcriptomic methods have opened up the field to rapid, sensitive and comprehensive assessment of microbiota occurring in different natural environments [1], [2], including the gut of various species [3], [4], diseased individuals [5], and apparently healthy tissues [6], [7].

Social insects (ants, bees, wasps and termites) live in cooperative colonies, often in high densities and with closely related individuals. They engage in contact behaviors such as trophallaxis (transfer of food or other fluids through mouth-to-mouth or anus-to-mouth feeding) and allogrooming. Crowded living conditions and contact behaviours allow disease to spread rapidly through colonies, and since nest mates are often related they tend to be genetically susceptible to the same pathogen [8], [9]. Compared to solitary insects, social insects are hence believed to be under increased pathogen pressure. In response, social insect have an expanded multilevel immune repertoire. These include highly conserved molecular defense pathways such as Toll, imd, Jak/STAT, JNK and RNAi [10]–[14], and mechanical and humoral cellular responses such as phagocytosis, nodule formation, encapsulation and antimicrobial peptides [8] that are shared with other holometabolous insects. Furthermore, and in contrast to solitary insects, social insects also have social immunity, i.e. individuals mount immune responses for the benefit of others [9], [15]–[17]. Social immunity in ants and other social insects include the use of specialized glands [8], and a variety of behaviours such as allogrooming to remove parasites from one another [18], task specialization [19], [20] and nest maintenance [9]. In spite of their importance for the evolution of immunity, natural pathogen communities have been assessed in detail only for honey bees [5] and one species of ant, the red invasive fire ant *Solenopsis invicta* [21] (and references therein).

Another aspect of social contact behaviours such as trophallaxis is that they provide opportunities for frequent transmission of gut bacteria, which is relatively rare in non-social insects [22], [23]. Consequently, social insects have some of the most distinctive and consistent gut microbial communities known among the insects. These communities have evolved to provide specialized beneficial functions in nutrition and protection for their host [22], [23]. Gut microbiota has been well characterized in the honey bee, and broadly characterized in some 60 species of ant [24]–[27]. These studies show taxonomic as well as dietary influence on the composition of gut bacterial communities in ants [22], [26].

*Formica exsecta* is a common ant species with a distribution range spanning most of Northern Eurasia [28]. It builds nests reaching diameters and depths up to 1–1.5 m, which consist of a soil core overlaid by needles, grass, and small sticks [29], [30]. Nests that reach maturity are relatively long lived (upto 20 years), however many fail to reach maturity, hence the average life span is six to seven years [31], [32]. It is an omnivore, feeding mainly on small insects and honey dew collected from aphids [33]–[35]. Eggs developing into sexuals are laid in May and mature to adults in June/July. Eggs destined to develop into new workers are laid intermixed with sexual-destined eggs and directly after them. Workers overwinter as adults and live for about one year, whereas male lifespan is restricted to a few weeks [29]. With regards to potential infectious agents or symbionts, previous studies have shown a high population/nest prevalence of the intracellular parasite *Wolbachia pipientis* in several populations of *F. exsecta*, although the rate of individual infection is unknown [36]–[38]. Several species of mites have been associated with this and other *Formica* ants [19], [39], [40]. The bacterial gut communities of this ant species have not been investigated.

This study joins several other recent publications where transcriptomic or genomic approaches have been used to identify sequences with homology to pathogenic and natural communities on and within species. Following the generation and assembly of an *F. exsecta* transcriptome, we utilized only those contigs that were found to not be of ant origin with the aim of identifying sequences with homology to potential intracellular pathogens and symbionts, including viruses, bacteria, and fungi; and biota closely associated with the ants and their environment. There were sequences with homology to two viruses, to several potential intracellular parasites (including *Wolbachia,* and candidates such as *Arsenophonus*), to potential beneficial symbionts (such as *Entomoplasma, Burkholderia* and yeasts), to six species of mite and to a range of common environmental fungi and bacteria.

## Methods

### Sampling and Sample Preparation

*Formica exsecta* queens, workers, males and cocoons developing to these castes were collected from six localities (three island localities: Rovholmarna, Joskär, Furuskär, and three mainland populations: Harparskog, Prästkulla, Ingå) spanning an area of approximately 50 km^2^ (Table S1). These islands are relatively pristine and uninhabited, and form part of a nature conservancy area in which agricultural activities, including apiculture, are strictly forbidden. The mainland localities were sampled in recently (<10–30 yrs.) clear-cut economical forest or meadowland close to small villages. No specific permission was required for sampling these ants. This species is not endangered or protected by Finnish or international laws. Finnish land legislation permits everyone the right to access all land without permission of the owner (Everyman’s right).

Established colony queens and overwintered workers (hereafter old queens and workers) were sampled straight from the field colonies in late April and early May 2011. They were immediately transported to the laboratory and frozen alive at −80°C. Cocoons were maintained in the laboratory until sex and caste could be reliably assessed from external morphology and size, and then divided into three developmental classes: young (white cuticle and eyes), intermediate (white cuticle but pigmented eyes), and old (pigmented cuticle and eyes). A proportion of the cocoons eclosed to new queens, males and workers. Samples were frozen as above at the appropriate developmental stage. For the purposes of this study we used the three castes: queens, workers and males. For the workers and queens we used three age classes: old, new and cocoon. New males and male cocoons were analyzed together due to the low sample numbers. Note that the cocoons were not analyzed by developmental stage. Table 1 lists detailed numbers of colonies, individuals, castes, ages and developmental classes.

**Table 1:** Number of individuals pooled in each of the total of 28 libraries sequenced by FIMM or BGI.

Caste/sex	Stage	FIMM	BGI
**Queen**	Old overwintered	6 (6)	4 (4)
	New	5 (10)	4 (8)
	Old cocoon	8 (4)	6 (3)
	Intermediate cocoon	8 (4)	6 (3)
	Young cocoon	8 (4)	6 (3)
**Worker**	Old overwintered	18 (6)	30 (8)
	New	10 (5)	15 (6)
	Old cocoon	8 (4)	6 (3)
	Intermediate cocoon	8 (4)	6 (3)
	Young cocoon	8 (4)	6 (3)
**Male**	Adult	3 (3)	3 (3)
	Old cocoon	3 (3)	3 (3)
	Intermediate cocoon	3 (3)	3 (3)
	Young cocoon	3 (3)	3 (3)

Castes and developmental classes of *F. exsecta* are given together with number of source colonies in parentheses.

The nests were not directly assessed for disease symptoms, however all nests were sampled by experienced researchers who noted no obvious signs of disease with regards to nest appearance, individual ant appearance and general worker behaviour. It was noted that many individuals collected in the field or housed in the laboratory carried mites that likely originated from the field colonies.

Prior to RNA extraction, samples were rinsed in 96% ethanol and any unspecific material, including mites, was removed under a microscope. Total RNA for RNA-seq library construction was extracted from whole queens, workers, males, and cocoons using TriSure reagent (Bioline), following the manufacturer’s protocol. Each sample was extracted individually, high quality RNA was determined by the presence of an 18S rRNA peak using the Agilent 2100 bioanalyzer, and RNA was pooled after extraction into the respective libraries (Table 1) based on caste and stage so that each RNA sample had equal representation in the pool. The exception to this was the library of old workers sequenced in BGI, which was normalized according to populations (not individuals) due to difficulties in obtaining high quality RNA. The final number of ants that were used was 209 individuals from 59 colonies.

The mRNA was selected from the total RNA (50–80 ug) pools using two rounds of Poly-A-Purist MAG kit (Ambion) and/or custom assays of library providers (see below). The first set of libraries was constructed by the Finnish Institute for Molecular Medicine (FIMM; University of Helsinki, Finland). In short, after the second round of poly-A selection and DNAse treatment cDNA was fragmented and sizes 200–500 bases were selected to construct Pair-End sequencing Illumina libraries. The second library set was constructed in the same manner by the Beijing Genomic Institute (BGI, China) except selecting only approximately 200 base fragments. Sequencing of the first set of libraries was conducted by FIMM (PE-99) and the second by BGI (PE-91), in both cases using two lanes of Illumina HighSeq 2000. The raw reads of the meta-transcriptome will be available from 2014-08-29 on GenBank (http://www.ncbi.nlm.nih.gov/genbank/) under bioproject ID PRJNA213662, sample accession numbers: SAMN02297446, SAMN02297447, SAMN02297448, SAMN02297449, SAMN02297450, SAMN02297451 and SAMN02297452.

### Basic Bioinformatic Analyses and NCBI BLAST Searches

An initial quality check of the raw fastq data was done by assessing the phred score (or Q-value, ranging from 0–40, where 40 denotes the highest quality) and trimming the low quality bases using Fastx toolkit v. 0.0.13 [41]. Transcriptome assembly from the trimmed reads was performed with the software Trinity (Release 2012-05-18, [42]). An initial transcriptome (IT) assembly was performed, followed by realignment of the original transcripts to the IT. This was a quality control step for all transcripts which ensured that at least five (forward and reverse) paired-end reads were used and removed transcripts which had very low reads per kilobase per million reads (RPKM, see below). Only transcripts with a confirmed minimum of five paired-end reads were used for BLAST searches and mapping against protein sequences from *Harpegnathos saltator, Camponotus floridanus* [43], *Atta cephalotes* [44], *Linepithema humile* [45], *Pogonomyrmex barbatus* [46], *S. invicta* [47] and *Acromyrmex echinatior* [48]. Full details of the final ant transcriptome assembly will be published elsewhere (Trontti et al. unpublished data). The reads that did not match to any ant genomes were BLAST searched against the virus, fungal, protozoan and bacterial genome databases at NCBI (BLAST version 2.2.26+). As cut off criteria in the BLAST searches we used a minimum alignment length of 100nt, E-value 0.001 a word size of 11, and a minimum of 70% sequence identity. Identified microbiota were then BLAST searched back against the *F. exsecta* transcriptome. By doing this we ensured that more than one gene in the identified microbiota was active so that sequence matches did not just represent single highly conserved genes/domains.

Expression values for the GenBank matches were calculated by aligning and counting all reads of each library, utilizing both FIMM and BGI data. From these, RPKM values (expression value in RNASeq) were calculated as follows: RPK/(total number of reads/1000000), where RPK = number of mapped reads/length of transcript in kb (transcript length/1000). This method quantifies gene expression from RNA sequencing data by normalizing for total read length and the number of sequencing reads. We give a total expression value, calculated over all ages and castes in Tables 2–4, and a breakdown of expression values by caste, age class and sequencing provider in Table S2. Note that the number of individuals from each age class and caste varied, from 3 to 30. The samples were pooled by sex and age-class (see above) prior to transcriptome expression in order to obtain sufficient amounts of RNA, hence the libraries were not analyzed by locality. Aware that only a fraction of microbiological diversity is described and/or present on GenBank, we chose to conservatively identify microbiota to genus level.

**Table 2:** Sequences matching microbes, and a mite, associated with *F. exsecta*, identified from GenBank BLAST searches.

Function	Kingdom	Phyla	*Genus*	Expressionvalue	Description
**Intracellular**	Virus	*Discistovirus*	*Dicistrovirus*	4945.261	Similar to IAPV, ABPV and KBV in honey bees (*Apis mellifera*).
		*Iflavirus*	*Iflavirus*	2984.429	Similar to DWV in honey bees.
	Bacteria	*Proteobacteria*	*Wolbachia_978*	321.331	Obligate endoparasite, widespread in arthropods, including *F. exsecta.*
		*Proteobacteria*	*Wolbachia_416*	3510.295	A second strain of *Wolbachia*, known from previous studies on *Formica* ants.
	Fungi*	*Microsporidia*	*Encephalitozoon*	17.902	Unicellular parasites, related microsporidians (*Nosema* spp.) cause infection in bees.
	Fungi	*Basidimycota*	*Cryptococcus* ∧	9.771	Some members of this genus are parasitic – their effect on insects (if any) is unknown
**(Likely)** **Extracellular**	Bacteria	*Proteobacteria*	*Acidiphilium*	8147.115	Acidophile, widespread in soils.
		*Actinobacteria*	*Rhodococcus*	7949.379	Generalist soil genus.
	Fungi	*Ascomycota*	*Aspergillus* ∧	41.899	Pervasive in the environment, including indoor air, some opportunistic pathogens
			*Penicillium*	110.729	
			*Neurospora*	2888.620	
			*Gibberella*	11.147	Environmental, inlcuding plant pathogens
			*Podospora*	5.751	Plant pathogen
			*Eremothecium*	26.331	
			*Yarrowia*	28.028	
		*Basidiomycota*	*Ustilago*	5.080	
	Animalia	*Arthropoda*	*Varroa*	64.196	Varroa destructor, mite well known from honey bees
**Unknown**	Fungi	*Ascomycota (yeast)*	*Candida*	3.144	Often found as symbionts in insects, including ants. Common and diverse in soils.
			*Saccharomyces*	2.606	
			*Scizosaccharomyces*	4.035	
			*Zygosaccaromyces*	4.582	
			*Kluyveromyces* ** ∧	56.651	
			*Naumovozyma*	4.658	
			*Tetrapisispora*	2781.217	
			*Torulaspora*	48.677	

Total expression value represents the combined expression value for all ages and castes, from both FIMM and BGI data.

Intracellular microbes are known parasites or have at least one stage of their lifecycle inside the host. Extracellular microbes do not usually have lifecycles within hosts, but can in some cases be opportunistic pathogens. Unknown is used for yeast, these are pervasive in environments that contain moisture and carbohydrate sources, and may form symbiotic relationships with ants in their guts, or they can grow in the nest material, or be carried in via aphid dew that ants feed on. IAPV = Israel Acute Paralysis Virus, Acute Bee Paralysis Virus, KBV = Kashmir Bee Virus, DVW = deformed wing virus.

* current classification.

** Some *Kluyveromyces* are teleomorphs of *Candida.*

∧ Fungi that gave hits on both common and species specific genes in GenBank.

**Table 3:** Sequences matching eukaryotes of non-fungal origin identified from the 18s rRNA gene.

Phylum	Superorder	Order	“cohort”	genus	Expressionvalue	Genbank match
*Arthropoda*	*Acariformes*	*Sarcoptiformes*	*Astigmata*	*Acarus*	3.427	EU152495.1.1543
			*Astigmata*	*Ewingia*	7.687	EU152502.1.1702
			*Astigmata*	*Histiostoma*	73.472	GQ864328.1.1656
			*Astigmata*	*Tyroborus*	13.140	EF203768.1.1516
	*Parasitiformes*	*Mesostigmata*	*Dermanyssina*	*Holostaspis*	23.156	FJ911851.1.1592
				*Gaeolaelaps*	3.265	FJ911852.1.1593
			*Veigaiidae*	*Veigaia*	7.186	GQ864298.1.1627

GenBank match denotes the GenBank accession number of the closest GenBank BLAST match. Total expression value represents the combined expression value for all ages and castes, from both FIMM and BGI data.

**Table 4:** 16s rRNA sequence analysis.

Phylum	Subphylum	Genus	Expressionvalue	GenBankmatch	Comment
*Actinobacteria*	*Actinomycetales*	*Micrococcineae*	38.48	JQ659818	This phylum and genus are very common soil bacteria
			37.70	AY189308	Soil
			37.88	JQ659818	Soil
*Bacteroidetes*	*Sphingobacteriaceae*	*Pedobacter*	5.63	CP001681	Soil
*Firmicutes*	*Streptococcaceae*	*Streptococcus*	4.38	AB682354	Soil
	*Lactobacillaceae*	*Lactobacillus*	77.63	FN667112	Common in soils. Symbiotic with e.g. honey bees
*Proteobacteria*	*Acetobacteraceae*	*Saccharibacter*	67.51	JN846886	*Camponotus fragilis* gut bacteria.
			99.24	JN846886	
		*Kozakia*	96.64	JN846887	
	*Anaplasmataceae*	*Anaplasma*	3793.28	CP001391	*Wolbachia* was closest GenBank match
	*Burkholderiaceae*	*Burkholderia*	8.07	JF763863	Common soil bacteria. Often symbiotic with arthropods
			21.51	CP000571	
	*Enterobacteriaceae*	*Arsenophonus*	4.57	JN990929	Insect specialized intracellular clade
	*Moraxellaceae*	*Enhydrobacter*	46.32	EU706098	Soils/symbionts
		*Acinetobacter*	37.37	JQ435689	
	*Pseudomonadaceae*	*Azomonas*	78.38	JQ659356	Common in soils, also symbiotic or pathogenic in several taxa
		*Azomonas*	56.54	EU072020	
		*Pseudomonas*	110.73	HE586388	
*Tenericutes*	*Entomoplasmataceae*	*Entomoplasma*	50.24	U67946	Obligate intracellular microbes. Often in association with insects
			40.78	AF547212	
		*Mesoplasma*	43.20	HM996851	

Phylum, subphylum and genus from the RDP Classifier. The closest GenBank match is given with the total expression value from both FIMM and BGI data, together with a short description of the most likely habitat, derived from GenBank accessions and literature (further details in the discussion).

### Ribosomal RNA

To cast the net wider we also isolated ribosomal GenBank matches from the transcriptome, namely the 16S, 18S and 28S ribosomal subunit genes for analysis. Sequences matching 18S rRNA eukaryotes were identified using NCBI BLAST searches. The Ribosomal Database Project (RDP) Classifier tool (http://rdp.cme.msu.edu/, [49]) was used to to classify sequences corresponding to the 16S rRNA gene in bacteria and the 28S gene (Large Subunit (LSU)) in fungi. The RDP Classifier tool implements a naïve Bayesian Classifier to taxonomic classification based on these genes, using a minimum of 80% confidence of assignment. For bacteria, we also obtained the best match cultivated type strains from the Ribosomal Database [50], and aligned these sequences with those found from the transcriptome using Muscle as implemented in SeaView v. 4.4.1 [51]. A phylogenetic tree was constructed by PhyML, again as implemented in SeaView v. 4.4.1 (model choice GTR, 100 bootstraps). A complimentary analysis of the fungal large subunit (LSU) genes was not possible owing to lack of suitable type strains and data quality.

### Additional Analyses

#### Viruses

GenBank database searches revealed that ant-derived transcripts showed sequence similarity to viruses from the *Dicistroviridae* and *Iflaviridae* families (both virus families are insect-specific [52], [53]). The available genomes of all Dicistroviruses and Iflaviruses were obtained from GenBank, and aligned with the ant-derived virus transcripts using ClustalW [54]. A phylogenetic tree was constructed from the alignment using the Maximum Likelihood method as implemented in PHYLIP 3.68 [55], with 100 bootstraps.

Some insect viruses are spread by vectors, or have low host-specificity such as many of the honey bee viruses [56], [57]. Since there were many phoretic mites on the ants, and since there were sequences with identity to the honey bee mite *Varroa destructor* in our transcriptome data (see below) we ruled out *V. destructor* as a vector or ant food source by BLAST searching of another *F. exsecta* transcriptome, generated by Badouin et al. [58] and publicly available on www.antgenomes.org [59]. The samples for the Badouin et al. [58] transcriptome came exclusively from the *Varroa*-free island localities Furuskär and Joskär, which were also sampled for our study (see methods). We further BLAST searched this transcriptome for sequences with homology to the viruses genomes derived from our transcriptome data.

#### Wolbachia

Six different strains of *Wolbachia* have been associated with several *Formica* species in Finland and Europe [37], [38]. These strains do not appear to be specific to individual ant species, and concomitant infections of two or several strains commonly occur [37], [38]. We confirmed that the sequences in our contigs matched two of these previously identified strains, by performing an alignment of *wsp* genes (outer surface protein precursors) from the ant-derived transcripts to *Wolbachia wsp* sequences already reported in GenBank (GenBank accession numbers: AY101196-AY1011200 from *Formica exsecta* [37] and EF554317 from *Formica rufa* [38]). Alignment was performed using MUMMER 3.23. [60].

## Results

All reads were found to be of high quality (Q>20), but trimming reduced the average read length from 99 bp to 90 for FIMM data, and 91 to 85 for BGI data. The IT assembly and realignment led to the removal of 48.6% of the total transcripts, leaving 81407 transcripts. A total of 53504 of these contigs did not align with the seven ant genomes (*H. saltator, C. floridanus*, *A. cephalotes*, *L. humile*, *P. barbatus, S. invicta* and *A. echinatior*), annotated using protein sequences. Of those contigs, 5746 returned sequence matches for organisms entered on the individual GenBank databases.

### GenBank BLAST Searches

Sequences with identity to *Wolbachia* were expressed in many contigs and with high expression values (Table 2, Table S2). Combined expression values for the two strains were lowest in the old queens and workers, and highest in cocoons. Ant-derived transcripts showed homology with over 300 *Wolbachia* genes. Sequences with similarity to *Wolbachia* were also confirmed in the 16S rRNA data (see below). The ant-derived sequences matched strains wFex4 (alignment lenght: 357 bp, identity: 100, coverage: 59.2%) and wFex2 (alignment lenght: 599 bp, identity: 99.5, coverage: 99.83%). Sequences with homology to two soil/environmental prokaryotes, *Rhodococcus (Actinobacteria)* and *Acidiphilum (Proteobacteria)* were also expressed in all ages and castes from the ant-derived transcripts; the former is common in soils, the latter is an acidophile (Table 2, Table S2).

**Figure 1:**
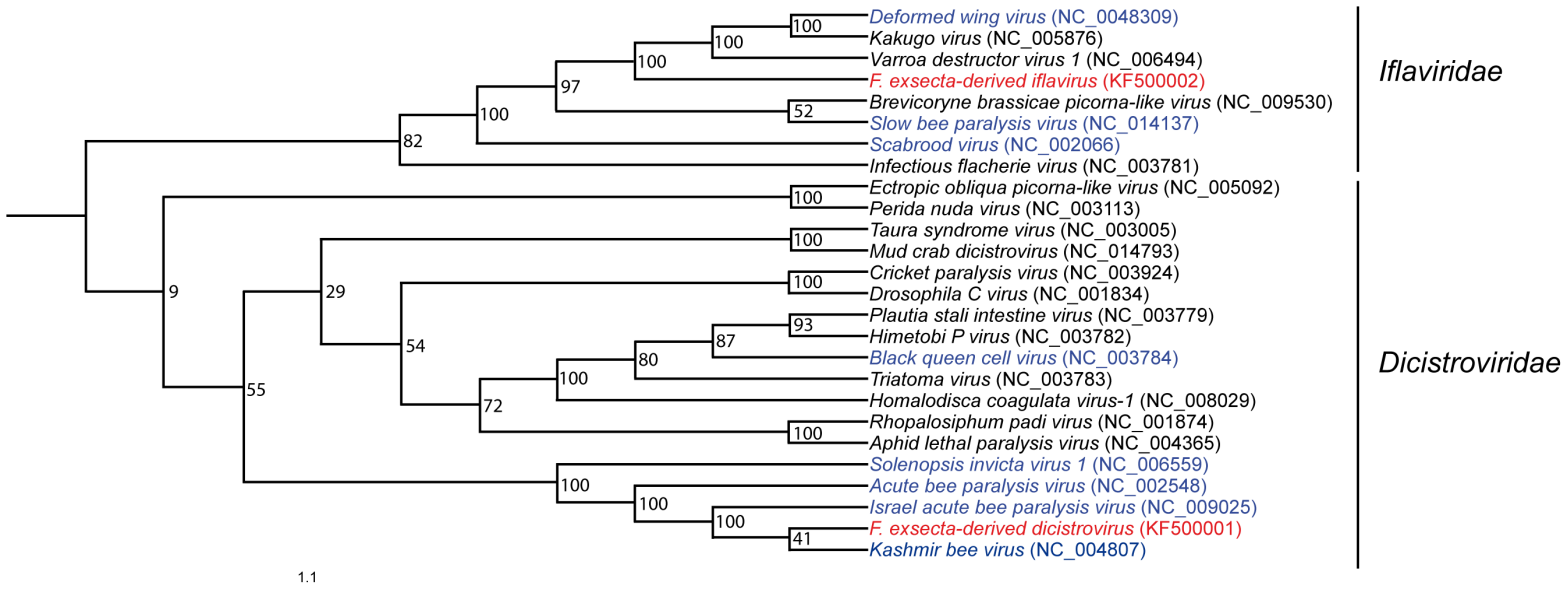
Phylogeny of the available genomes of the insect-specific *Discistroviridae* and *Iridoviridae* from GenBank. Social insect viruses are highlighted in blue and ant-derived assembled virus genomes from this study are highlighted in red.

Sequences with homology to one Dicistrovirus and one Iflavirus were present in the transcriptome data, and had very high expression values (Table 2, Table S2). Both *Iflaviridae* and *Dicistroviridae* belong in the (+) ssRNA viruses (+) sense RNA, with similarity to *Picornavirales*. Our phylogenetic analyses showed that the genome with homology to *Dicistroviridae* grouped with Dicistroviruses found in a species of ant and several species of bee, whereas the genome with homology to *Iflaviridae* grouped with Iflaviruses infecting several different insect species, including bees (Figure 1). The ant-derived Dicistrovirus had 75% nucleotide sequence similarity to the Kashmir Bee Virus, and the ant-derived Iflavirus had 70% nucleotide sequence similarity to the Deformed Wing Virus. The sizes of both virus genomes from the initial assembly (Dicistrovirus: 9554 bp, Iflavirus: 9160 bp) were consistent with genome sizes found in the other viruses in Figure 1 (range: ca 8000–10500 bp). Sequences matching the Discistrovirus were expressed in all ages and castes, with the highest expression value found in new workers, and the lowest expression in old queens. Sequences matching the Iflavirus were expressed in all castes and ages, with the highest expression value in new queens and the lowest in new workers (Table S2). Annotated virus draft genome assemblies are available under accession numbers KF500001 (Dicistrovirus) and KF500002 (Iflavirus) on GenBank. We identified the contig ‘isotig09016’ in the published *F. exsecta* transcriptome generated by Badouin et al., [58] which showed 99% identity with sequences yielding identity to the Iflavirus in our transcriptome.

Sequences with homology to seventeen species of fungi were present in the transcriptome data, seven of which were yeasts, two were common moulds (*Aspergillus, Penicillium*), six were plant pathogens, one a facultative endoparasite (*Cryptococcus*) and one an intracellular microsporidian from the genus *Encephalitozoon*. Since many of these fungi may share conserved genes we collapsed this list by removing sequences with homology only to genes shared with all other fungi (Table 2). This collapsed list included sequences with high similarity to only four species: *Aspergillus oryzae, A. niger, Kluyveromyces* and *Cryptococcus,* which were expressed in all castes and ages.

In spite of careful removal of mites from the ants, there were sequences with identity to *V. destructor* genes present in the transcriptome data (Table 2, Table S2) Notably, sequences matching *V. destructor* were not present among the 18S rRNA sequence data generated from our transcriptome data (see below), nor from Badouin et al.’s [58] transcriptome. *Varroa destructor* is the sole mite species that has had its genome sequenced, so similar genes and sequences from other species of mites will most likely also show homology with *V. destructor* sequences submitted to GenBank. The expression value was highest in new queens, and lowest in old workers, and the expression values were overall similar to expression patterns for total mite 18s rRNA sequences.

### Ribosomal RNA BLAST Searches and RDP Classifier Results

The 18S rRNA sequences yielded matches to genes of seven mite species (Table 3). Both mite super-orders, *Acariformes* and *Parasitiformes*, were represented, sorting into suborders *Sarcoptiformes; Astigmata* (four members) for the former and *Mesostigmata; Dermanyssina* and *Mesostigmata: Veigaiidae* for the latter (three members). Sequences yielding identity to *Histiostoma* showed the highest expression level overall (Table 3) and were expressed in all castes and age classes with the exception of old queens (Table S2), with the highest expression value in new queens. Sequences yielding identity to *Veigaia* had relatively low expression values, however they were expressed in all ages and castes (Table 3, Table S2). Total expression values for sequences matching mite 18S rRNA was highest in new workers and queens, and lowest in old queens and worker cocoons. No sequences with similarity to 18S rRNA mite genes were detected in the *F. exsecta* transcriptome generated by Badouin et al. [58].

**Figure 2:**
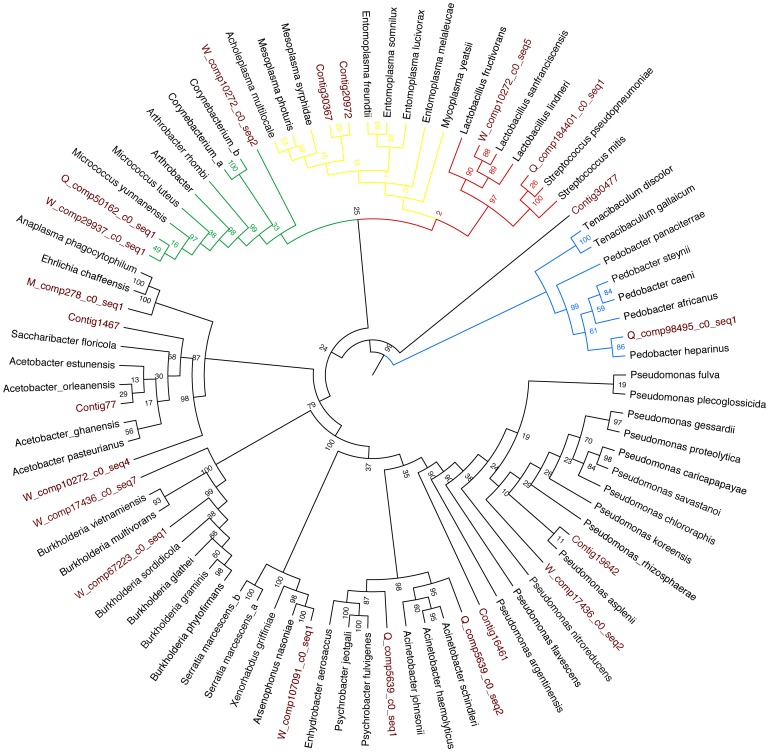
Phylogenetic tree of bacterial 16S rRNA sequences, with closest type strains obtained from the Ribosomal Database project in black font. Ant-derived contig IDs are given in red font. Red clade = *Firmicutes*, blue clade = *Bacteroidetes*, black clade = *Proteobacteria*, yellow clade = *Tenericutes*, green clade = *Actinobacteria.*

A total of 249 sequences that exhibited identity to bacterial 16S rRNA were retrieved from the transcriptome data and entered into RDP Classifier. Of these, 21 were retained for further analyses. The excluded matches failed to meet RDP criteria by having sizes less than 200 bp or failing alignment (188 sequences), 37 sequences were classified as bacteria but no further classification was possible, three were classified as chloroplasts, and three had confidence intervals below 80% at more than one stage of classification. NCBI BLAST matches and RDP classification mostly agreed, in case of disagreement NCBI BLAST results were chosen as the default (Table 4). Among the retained 21 sequences were matches to *Firmicutes, Tenericutes, Actinobacteria* and *Proteobacteria* (Table 4). A phylogenetic tree of these sequences and their closest cultivated type strains obtained from RDP is shown in Figure 2. Overall, the bootstrap values showed that the tree was not very robust in some of its deeper branches (which was expected considering that the tree spans deep phylogenetic divisions within bacteria). However, some of the sequences isolated from the ant transcriptome resolved well at the tips of the tree with particularly close matches to the type strains for *Lactobacillus, Micrococcus, Arsenophonus,* and *Pseudomonas*. Furthermore, type strains for *Entomoplasma, Burkholderia, and Wolbachia* were also good matches to our sequence data.

Among the 21 16S rRNA sequences retained from the RDP classifier there were sequences with homology to bacteria whose life cycles are either completely within a host cell or require some stage of their life cycle within a host cell (hereafter intracellular). These were: *Proteobacteria* (*Wolbachia*), *Enterobacteriacae* (*Arsenophonus),* and *Tenericutes* (*Entomoplasma*). Sequences with identity to *Acetobacteracaeae* matched most closely to gut bacteria isolated from the ant *Camponotus fragilis*, and may also be intracellular. Total expression values when combined by genus showed that sequences with identity to *Wolbachia* had the highest value, followed by sequences yielding identity to *Acetobacteraceae* and sequences with homology to *Entomplasmataceae.* Sequences matching *Arsenophonus* did not have high expression values and were less encountered than the above genera (Table 3.) Sequences matching *Wolbachia* and *Acetobacteraceae* were expressed in all castes and ages (*Wolbachia* expression patterns as above), whereas sequences matching *Arsenophonus* were not expressed in new queens and new workers (Table S2). The remaining sequences matched bacteria from subphyla commonly found in soils (subphyla *Actinomycetales*, *Sphingobacteriaceae, Streptococcacae, Lactobaciallaceae, Burkholderiacae, Moraxellaceae* and *Pseudomonadaceae).* Several members of these subphyla are capable of a beneficial or pathogenic symbiotic lifestyle with insect hosts, in particular *Lactobaciallaceae*, *Burkholderiaceae* and *Pseudomonadaceae.* Sequences matching these latter three genera were expressed in all castes and ages (Table S2).

Fifty-four LSU sequences were retrieved from the transcriptome data. Of these, eight failed to be classified as fungi using the RDP classifier, another 35 were classified as fungi, but no further taxonomic classification was possible, leaving only eight sequences that could be classified with some confidence (>80%) to species level. Of these, four sequences were classified as *Basidiomycota*, species *Hohenbuehelia* (wood decaying fungi), and the remaining sequences were classified as Ascomycota, two classifying as *Plectosphaerella* (plant pathogen) and two as *Parmotrema* (lichen) (Table S3).

## Discussion

Sequences with identity to a wide range of microbes, and some species of mite, were retrieved from within and on the ant *Formica exsecta*, by analysing data of non-ant origin from transcriptome assemblies. These included sequences yielding identity to two viruses, two *Wolbachia* strains, several potential intracellular symbionts (*Arsenophonus*, *Lactobacillus* and *Microsporidium*) and potentially opportunistic pathogens (*Aspergillus* spp. and *Pseudomonas* spp.). Since transcriptome data is generated from RNA, the sequence matches most likely represent some of the most active microbes associated with the ant *F. exsecta*.

The successful and expected retrieval of sequences matching *Wolbachia* from many contigs and among the 16S rRNA sequence data attests to the feasibility of this approach. Sequences matching *Wolbachia* showed high levels of identity with two strains that commonly infect Eurasian and Finnish populations of several closely related *Formica* wood ants, including *Formica exsecta* [36]–[38], [61]. *Wolbachia* is a very common and widespread intracellular symbionts of insects [62], including ants [36]. It usually shows no noticeable adverse effect but occasionally affects its host negatively [63]. It is readily horizontally and vertically transmitted within and between *Formica* species, and has not been associated with any detrimental effects in *F. exsecta* [38], [61], [64]. Expression of sequences matching *Wolbachia* appeared to decrease with age, in line with previous results on *F. exsecta* by Keller et al. [61]. The mechanism and significance of this pattern remains unknown.

In addition to sequences yielding identity to *Wolbachia,* there were sequences with homology to other intracellular microbes. These were to the fungi *Microsporidia,* and to bacteria belonging to the *Enterobacteriacae* (*Arsenophonus),* the *Entomoplasmatales*, and the *Aceterobacteraceae* (*Saccharibacter). Microsporidia* are unicellular, fungal intracellular parasites. Present as spores in the soil, they are ingested and replicate in a wide range of hosts, including insects [65]. Well-studied microsporidians in social insects include *Kneallhazia solenopsae* [66]–[68] and *Vairimorpha invictae* [69] that both infect the fire ant *Solenopsis invicta*. *Kneallhazia solenopsae* cause substantial brood reductions, queen debilitation and premature death of infected queens [70], [71], and *V. invictae* cause significant reduction in nest growth and higher sensitivity to starvation [72], [73]. Both of these microsporidia are present in larvae and adults. Bacteria belonging to the *Enterobacteriacae* (*Arsenophonus),* and the *Entomoplasmatales* (particularly *Spiroplasmas*) are known reproductive parasites among various insect species, including social Hymenoptera [27], [62]. Screening across taxa and species has revealed that these bacteria are usually found in lower prevalence than *Wolbachia* [62], which expression values suggested was also the case for our data. *Arsenophonus* is best known from its association with the parasitic wasp *Nasonia vitripennis*, where it causes elevated death rates among male pupae [74], however recent research suggest that it is one of the richest and most widespread genera of insect associates, with effects ranging from male-killing to symbiosis [75]. *Entomoplasmatales* are also primarily insect associated bacteria, found in a diversity of roles, again ranging from male-killing to beneficial symbiosis [76]. *Entomoplasmatales* have been found in ant guts, forming a host-specific clade among the army ants [76], and *Spiroplasmas* were recently suggested to be prime candidates as a beneficial symbionts in two *Solenopsis* species [27]. Several 16S rRNA sequences had their nearest GenBank match to bacteria found in the gut of another ant, *Camponotus fragilis* (GenBank IDs: JN846886 and JN846887). These were classified as *Aceterobacteraceae* by the RDP classifier. *Aceterobacteraceae* have been found in honey bee guts, and one such bacteria, *Saccharibacter,* has also been isolated from pollen, and may hence represent bacteria taken up through foraging [77]. The expression patterns in our data for sequences matching potential intracellular microbes varied and included: present in all ages and castes (e.g. *Acetobacteraceae),* absent only in the old age classes *(Entmoplasmatales),* present in only in new queens and workers *(Microsporidia),* and no discernable pattern (*Arsenophonus).* Both beneficial and pathogenic intracellular microbes may predominantly occur in certain age classes [22], hence it is impossible to derive any definitive indication of role from this data.

Two apparent entire RNA virus genomes were assembled from the *Formica exsecta* transcriptome. Both were positive-sense single-stranded RNA viruses, one belonging to the class Iflaviridae and the other to the Dicistroviridae. These viruses grouped phylogenetically with the KBV, ABV and IAPV viruses that plague apiculture [78], [79], and with the Solenopsis invicta virus-1 that infects *S. invicta* [80]. These viruses are rapidly evolving due to their lack of repair mechanisms and often the same virus can infect several different insect hosts [56], [57]. The virus genomes assembled from the *F. execta* transcriptome appear rather dissimilar to honey bee viruses, with no more than 75% nucleotide sequence identity to the phylogenetically closest honey bee viruses. Hence, these viruses possibly warrant classification as new viruses, however such verification and analysis (e.g. electron microscopy, negative strand qPCR and epidemiology) is beyond the scope of this study. None of the ants sampled here showed any obvious symptoms similar to those reported for bees with high levels of infection of Dicistroviruses (trembling, paralysis) or Iridoviruses (discolouring), and the pathology and extent of infection in natural ant populations are unknown. Several viruses infecting ants have been described: Solenopsis invicta virus-1, 2 and 3 characterised for *Solenopsis invicta* [80]–[82], and a *Rhabdovirus*, a picorna-like virus and a virus of uncertain classification recently identified from transcriptome data in the Caribbean Crazy ant, *Nylanderia pubens* [83]. Viruses from several different classes hence appear common among ants.

One route for RNA viruses to enter insects is through ingestion, e.g two ant species, *Formica rufa* and *Camponotus vagus* were found to have ingested honey bee viruses by feeding on dead *Varroa* in honey bee hives [84], and Valles et al [85], reported sequences matching a cricket virus gene in ant-derived expressed sequence tag data, thought to originate from laboratory antfeed. In our transcriptome there were sequence matches to *V. destructor,* a mite that attacks the honey bee (*Apis mellifera*) [5], [86], However, there were no sequences matching *Varroa* 18S rRNA, a gene which is used for mite species identification [87]. Instead there were 18S rRNA sequence matches to six species of mite from genera with known ant associations [43], [93], [94]. Moreover, expression patterns of sequences matching total mite 18S rRNA and expression values for those sequences matching *V. destructor* were very similar. Hence, sequences matching *Varroa* were likely an artefact as genes from other species of mites are likely to match *V. destructor,* currently the only mite genome available in GenBank. No sequences matching Varroa 18S rRNA were present in the second transcriptome generated from *F. exsecta* ants living in habitat where apiculture is forbidden, yet sequences matching the Iflavirus was present. Sequences matching the Dicistrovirus were not present in this second transcriptome, however this is not surprising since the lowest expression values for sequences matching this virus in our data was in old workers (the age and caste sampled for the Badouin et al. transcriptome).

Nevertheless, mites (Acari) are ubiquitous and diverse in *Formica* wood ant nests [39]. They associate with ants mainly for dispersal (phoresy), or for feeding on bacteria and detritus in the nest or on the ants [40]. Since it is the task of adult workers to forage we would expect the the highest expression values for sequences matching both mites and viruses in this caste and age class if either were indeed ingested. If the viruses were transmitted by mites, we would expect some similarity in expression patterns of the sequences with homology to mite genes and those with homology to viruses. To the contrary, old workers had relatively low expression values for 18S rRNA sequences matching any mites, high expression levels for sequences matching the Iflavirus and low expression values for sequences matching the Dicistrovirus. The highest expression value for 18S rRNA sequences matching mites was found in new queens, which had low expression values for sequences matching the Iflavirus, and relatively high expression values for sequences matching the Dicistrovirus. In short, there appears to be no association in this data between sequences matching mites and expression of sequences matching either of the viruses.

Some of the microbes for which there were sequence matches quite possible have roles both as beneficial ant symbionts and in the soil or nest environments. Yeasts, for example, are common in the environment [88] and are unlikely insect pathogens [89]. Many insects are dependent on yeasts as sources of sterols and vitamins. A study in *S. invicta* found several yeasts present in the nest and guts of both larvae and workers. These provided nutrients during overwintering, thereby increasing nest survival and productivity [90]. Among bacteria, *Burkholderiales* and *Pseudomodales* are commonly found in ant guts and are thought to be beneficial symbionts [26], [27], however they are also widespread in soils and may simply have been ingested by *F. exsecta*. *Lactobacillus* are commonly found in acidic environments such as composts, yet some species are also well characterised beneficial gut symbionts in e.g. honey bees and bumble bees [77] and have also been recovered from ant guts, although any functional role in ants is unknown [26]. Sequences matching *Burkholderiales, Pseudomodales*, *Lactobacillus* and yeasts were expressed in all age classes, hence intra- or extracellular origin cannot be determined in this study.

Common soil bacteria or fungi may also act as opportunistic pathogens. There are several known generalist pathogens in the genus Aspergilli, but none of the species are exclusively entomopathogenic. *Aspergillus ochraceaus* is an example of a common environmental Aspergillus with entomopathogenic properties. This fungi infects the ant *Atta bisphaerica* resulting in about 50% mortality [91]. Another potentially pathogenic fungi for which there were sequence matches in our data is *Cryptococcus*, which is widely distributed in soils and best known for causing infection in immuno-compromised humans [92]. However, strains of *Cryptococcus* have been isolated from dead insects or insect chaff [92], although their role in insects is unknown.

The remaining sequences matched mainly fungal plant pathogens and common soil bacteria. These may be derived from ant feed or nest material. There were very few sequences matching saprotrophic wood or plant decaying fungi considering that *F. exsecta* mounds are made of plant material (including needles and other material from coniferous trees). The analysis of fungal LSU genes showed the difficulty of molecular identification of fungal species [93] compared to the bacterial classification scheme based on 16S rRNA. Unexpectedly, the entomopathogenic fungi *Metarhizium anisopliae* and *Beauveria bassiana,* common in soil samples in Finland [94], and known to infect ants [95], did not match sequences in the transcriptome data. Ants almost certainly carry fungal spores into the nest, however if these fail to germinate in the dry and warm environment of the nests, or on the ants themselves, they will not be detectable in transcriptomic data.

Two factors are likely to have affected our results. Firstly, a poly-A treatment of the RNA was performed to reduce the non-eukaryotic content in the samples, and secondly, we applied strict criteria for the bioinformatic analysis. As a result, we fully expect that there are even more microbes and other taxa associated with these ants. Certain trends were apparent and we are confident that the biota presented here is truly associated with, and of importance, in *F. exsecta*. Similar to *S. invicta* (in its native range) [21], *F. exsecta* is infected by *Wolbachia* and may be infected by several viruses and possibly also microsporidia. Transcriptome data derived from *F. exsecta* showed sequence matches to *Burkholderiales, Lactobacillus Acetobacteraceae* and *Pseudomodales* which are bacterial genera that often form part of the highly distinct bacterial gut communities in social insects [26], [77]. There were also sequence matches to common reproductive parasites, of social as well as solitary insects, such as *Entomoplasmatales* and *Arsenophonus.* Our study provides a starting point for further research to establish which of these potential associates are neutral elements, random benefactors, pathogens, or have decisive positive effects on the fitness of *F. exsecta* ants and their nests.

## Supporting Information

Table S1 **Geographic localities, numbers of** ***F. exsecta*** **nests sampled and number of individuals from those nests.**
(DOCX)

Table S2 **Expression values by sex and caste for all sequence matches in** **Tables 2** **–** **4** **, from both sequencing providers (Beijing Institute of Genomics, China, (BGI) and Finnish Institute of Molecular Medicine, Finland (FIMM).**
(DOCX)

Table S3 **RDP classifier classification of the fungal large subunit (LSU) gene sequences.**
(DOCX)

## References

[1] Damon C, Lehembre F, Oger-Desfeux C, Luis P, Ranger J, et al (2012) Metatranscriptomics reveals the diversity of genes expressed by eukaryotes in forest soils. PLoS ONE 7: e28967. 22238585 10.1371/journal.pone.0028967 PMC3253082

[2] Scott JJ, Budsberg KJ, Suen G, Wixon DL, Balser TC, et al (2010) Microbial community structure of leaf-cutter ant fungus gardens and refuse dumps. PLoS ONE 5: e9922. 20360970 10.1371/journal.pone.0009922 PMC2847949

[3] Moran NA, Hansen AK, Powell JE, Sabree ZL (2012) Distinctive gut microbiota of honey bees assessed using deep sampling from individual worker bees. PLoS ONE 7: e36393. 22558460 10.1371/journal.pone.0036393 PMC3338667

[4] Gosalbes MJ, Durbán A, Pignatelli M, Abellan JJ, Jiménez-Hernández N, et al (2011) Metatranscriptomic approach to analyze the functional human gut microbiota. PLoS ONE 6: e17447. 21408168 10.1371/journal.pone.0017447 PMC3050895

[5] Cox-Foster DL, Conlan S, Holmes EC, Palacios G, Evans JD, et al (2007) A metagenomic survey of microbes in honey bee colony collapse disorder. Science (New York, NY) 318: 283–287. 10.1126/science.1146498 17823314

[6] Wittekindt NE, Padhi A, Schuster SC, Qi J, Zhao F, et al (2010) Nodeomics: pathogen detection in vertebrate lymph nodes using meta-transcriptomics. PLoS ONE 5: e13432. 20976145 10.1371/journal.pone.0013432 PMC2956653

[7] Moore RA, Warren RL, Freeman JD, Gustavsen JA, Chénard C, et al (2011) The sensitivity of massively parallel sequencing for detecting candidate infectious agents associated with human tissue. PLoS ONE 6: e19838. 21603639 10.1371/journal.pone.0019838 PMC3094400

[8] Schluns H, Crozier RH (2009) Molecular and chemical immune defenses in ants (Hymenoptera: Formicidae). Myrmecol News 12: 237–249.

[9] Cremer S, Armitage SAO, Schmid-Hempel P (2007) Social immunity. Curr. Biol. CB 17: R693–702. 17714663 10.1016/j.cub.2007.06.008

[10] Brennan C, Anderson K (2004) *Drosophila*: the genetics of innate immune system recognition and response. Annu. Rev. Immunol. 22: 457–483. 10.1146/annurev.immunol.22.012703.104626 15032585

[11] Viljakainen L (2008) Evolutionary genetics of immunity and infection in social insects University of Oulu, Finland.

[12] Siva-Jothy MT, Moret Y, Rolff J (2005) Insect immunity - an evolutionary ecology perspective. Adv In Insect Phys 32: 1–48.

[13] Schluns H, Crozier YC, Crozier RH (2008) Sequence evolution in bulldog ants (Myrmeciinae). Proceedings of the 4th European meeting of the International Union for the Study of Social Insects. La-Roche-en-Ardenne, Belgium. p. 65.

[14] Simola DF, Wissler L, Donahue G, Waterhouse RM, Helmkampf M, et al (2013) Social insect genomes exhibit dramatic evolution in gene composition and regulation while preserving regulatory features linked to sociality. Genome Res 23: 1235–1247. 23636946 10.1101/gr.155408.113 PMC3730098

[15] Cotter SC, Kilner RM (2010) Personal immunity versus social immunity. Behav. Ecol. 21: 663–668.

[16] Cotter SC, Topham E, Price AJP, Kilner RM (2010) Fitness costs associated with mounting a social immune response. Ecol. Lett. 13: 1114–1123. 10.1111/j.1461-0248.2010.01500.x 20545735

[17] Cremer S, Sixt M (2009) Analogies in the evolution of individual and social immunity. Philos. Trans. R. Soc. Lond., B, Biol. Sci. 364: 129–142. 10.1098/rstb.2008.0166 PMC2666697 18926974

[18] Oi DH, Pereira R (1993) Ant behavior and microbial pathogens (Hymenoptera: Formicidae). The Florida Entomologist 76: 63–74.

[19] Schmid-Hempel P (1998) Parasites in social insects. Princeton: Princeton University Press.

[20] Bourke AFG, Franks NR (1995) Social evolution in ants. Princeton University Press.

[21] Yang C-C, Yu Y-C, Valles SM, Oi DH, Chen Y-C, et al (2010) Loss of microbial (pathogen) infections associated with recent invasions of the red imported fire ant *Solenopsis invicta*. Biol. Invasions 12: 3307–3318.

[22] Engel P, Moran NA (2013) The gut microbiota of insects - diversity in structure and function. FEMS Microbiol. Rev. 37: 699–735. 10.1111/1574-6976.12025 23692388

[23] Zientz E, Feldhaar H, Stoll S, Gross R (2005) Insights into the microbial world associated with ants. Arch. Microbiol. 184: 199–206. 10.1007/s00203-005-0041-0 16205909

[24] Stoll S, Gadau J, Gross R, Feldhar H (2007) Bacterial microbiota associated with ants of the genus *Tetraponera*. Biol. J. Linn. Soc. Lond. 90: 399–412.

[25] Russell JA, Moreau CS, Goldman-Huertas B, Fujiwara M, Lohman DJ, et al (2009) Bacterial gut symbionts are tightly linked with the evolution of herbivory in ants. Proc. Natl. Acad. Sci. U.S.A. 106: 21236–21241. 10.1073/pnas.0907926106 PMC2785723 19948964

[26] Anderson KE, Russell JA, Moreau CS, Kautz S, Sullam KE, et al (2012) Highly similar microbial communities are shared among related and trophically similar ant species. Mol. Ecol. 21: 2282–2296. 10.1111/j.1365-294X.2011.05464.x 22276952

[27] Ishak HD, Plowes R, Sen R, Kellner K, Meyer E, et al (2011) Bacterial diversity in *Solenopsis invicta* and *Solenopsis geminata* ant colonies characterized by 16S amplicon 454 pyrosequencing. Microb. Ecol. 61: 821–831. 10.1007/s00248-010-9793-4 21243351

[28] Douwes P, Abenius J, Cederberg, Björn Wahlstedt U (2012) Nationalnyckeln till Sveriges flora och fauna - Steklar: Myror - getingar. *Hymenoptera: Formicidae - Vespidae*. Artdatabanken/SLU, Uppsala.

[29] Seifert B (2000) A taxonomic revision of the ant subgenus *Coptoformica* Mueller, 1923 *(Hymenoptera, Formicidae)* . Zoosystema 22: 517–568.

[30] Czechowski W, Radchenko A, Czechowska W (2002) The ants (*Hymenoptera, Formicidae)* of Poland. Museum and Institute of Zoology. Poland.

[31] Haag-Liautard C, Vitikainen E, Keller L, Sundström L (2009) Fitness and the level of homozygosity in a social insect. J. Evol. Biol. 22: 134–142. 10.1111/j.1420-9101.2008.01635.x 19127611

[32] Pamilo P (1991) Life span of queens in the ant *Formica exsecta* . Insectes Soc 38: 111–119.

[33] Werner P, Catzeflis F, Cherix D (1979) A propos du polycalisme chez *Formica (Croptoformica) exsecta* Nyl. In: Cherix D, editor. Ecologie des Insectes Socieaux. Lausanne, Switzerland: UIEIS Section Francaise. 115–126.

[34] Pisarski B (1982) Characteristique de *Formica (Croptoformica) exsecta* Nylander 1946. Memorabilia Zoologica 38: 38–30.

[35] Pisarski B (1982) Territoires et territorialisme de *Formica (Croptoformica) exsecta* Nyl. Memorabilia Zoologica 38: 31–51.

[36] Wenseleers T, Ito F, Van Borm S, Huybrechts R, Volckaert F, et al (1998) Widespread occurrence of the micro-organism *Wolbachia* in ants. Proc. Biol. Sci. 265: 1447–1452. 10.1098/rspb.1998.0456 PMC1689219 9721689

[37] Reuter M, Keller L (2003) High levels of multiple *Wolbachia* infection and recombination in the ant *Formica exsecta*. Mol. Biol. Evol. 20: 748–753. 10.1093/molbev/msg082 12679529

[38] Viljakainen L, Reuter M, Pamilo P (2008) *Wolbachia* transmission dynamics in *Formica* wood ants. BMC Evol. Biol. 8: 55. 10.1186/1471-2148-8-55 PMC2277377 18291041

[39] Lehtinen, Tapiola P (1987) Associations of uropodid, prodinychid, polyaspidid, antennophorid, sejid, microgynid and zerconid mites with ants. Entomologisk Tidskrift 108: 13–20.

[40] Eickwort GC (1990) Associations of mites with social insects. Annu. Rev. Entomol. 35: 469–488.

[41] Pearson WR, Lipman DJ (1988) Improved tools for biological sequence comparison. Proc. Natl. Acad. Sci. U.S.A. 85: 2444–2448. 10.1073/pnas.85.8.2444 PMC280013 3162770

[42] Grabherr MG, Haas BJ, Yassour M, Levin JZ, Thompson DA, et al (2011) Full-length transcriptome assembly from RNA-Seq data without a reference genome. Nat. Biotechnol. 29: 644–652. 10.1038/nbt.1883 PMC3571712 21572440

[43] Bonasio R, Zhang G, Ye C, Mutti NS, Fang X, et al (2010) Genomic comparison of the ants *Camponotus floridanus* and *Harpegnathos saltator*. Science. 329: 1068–1071. 10.1126/science.1192428 PMC3772619 20798317

[44] Suen G, Teiling C, Li L, Holt C, Abouheif E, et al (2011) The genome sequence of the leaf-cutter ant *Atta cephalotes* reveals insights into its obligate symbiotic lifestyle. PLoS Genet. 7: e1002007. 10.1371/journal.pgen.1002007 PMC3037820 21347285

[45] Smith CD, Zimin A, Holt C, Abouheif E, Benton R, et al (2011) Draft genome of the globally widespread and invasive Argentine ant (*Linepithema humile*). Proc. Natl. Acad. Sci. U.S.A. 108: 5673–5678. 10.1073/pnas.1008617108 PMC3078359 21282631

[46] Smith CR, Smith CD, Robertson HM, Helmkampf M, Zimin A, et al (2011) Draft genome of the red harvester ant *Pogonomyrmex barbatus*. Proc. Natl. Acad. Sci. U.S.A. 108: 5667–5672. 10.1073/pnas.1007901108 PMC3078412 21282651

[47] Wurm Y, Wang J, Riba-Grognuz O, Corona M, Nygaard S, et al (2011) The genome of the fire ant *Solenopsis invicta*. Proc. Natl. Acad. Sci. U.S.A. 108: 5679–5684. 10.1073/pnas.1009690108 PMC3078418 21282665

[48] Nygaard S, Zhang G, Schiøtt M, Li C, Wurm Y, et al (2011) The genome of the leaf-cutting ant *Acromyrmex echinatior* suggests key adaptations to advanced social life and fungus farming. Genome Res 21: 1339–1348. 21719571 10.1101/gr.121392.111 PMC3149500

[49] Wang Q, Garrity GM, Tiedje JM, Cole JR (2007) Naive Bayesian classifier for rapid assignment of rRNA sequences into the new bacterial taxonomy. Appl. Environ. Microbiol. 73: 5261–5267. 10.1128/AEM.00062-07 PMC1950982 17586664

[50] Cole JR, Wang Q, Cardenas E, Fish J, Chai B, et al (2009) The Ribosomal Database Project: improved alignments and new tools for rRNA analysis. Nucleic Acids Res. 37: D141–5. 10.1093/nar/gkn879 PMC2686447 19004872

[51] Gouy M, Guindon S, Gascuel O (2010) SeaView version 4: A multiplatform graphical user interface for sequence alignment and phylogenetic tree building. Mol. Biol. Evol. 27: 221–224. 10.1093/molbev/msp259 19854763

[52] Van Oers MM (2010) Genomics and biology of Iflaviruses. In: Asgari S, Johnson K, editors. Insect Virology. Caister Academic Press, Norfolk. 231–250.

[53] Christian P, Carstens E, Domier L, Johnson J, Johnson K, et al.. (2005) Dicistroviridae. In: Fauquet CM, Mayo MA, Maniloff J, Desselberger U, Ball LA, editors. Virus Taxonomy VIII. Elsevier Academic Press, San Diego. 783–788.

[54] Larkin MA, Blackshields G, Brown NP, Chenna R, McGettigan PA, et al (2007) Clustal W and Clustal X version 2.0. Bioinformatics. 23: 2947–2948. 10.1093/bioinformatics/btm404 17846036

[55] Felsenstein J (1989) Phylogeny Inference Package (Version 3.2). Cladistics 5: 164–165.

[56] Levitt AL, Singh R, Cox-Foster DL, Rajotte E, Hoover K, et al (2013) Cross-species transmission of honey bee viruses in associated arthropods. Virus Res. 176: 232–240. 10.1016/j.virusres.2013.06.013 23845302

[57] Singh R, Levitt AL, Rajotte EG, Holmes EC, Ostiguy N, et al (2010) RNA viruses in hymenopteran pollinators: evidence of inter-taxa virus transmission via pollen and potential impact on non-Apis hymenopteran species. PLoS ONE 5: e14357. 21203504 10.1371/journal.pone.0014357 PMC3008715

[58] Badouin H, Belkhir K, Gregson E, Galindo J, Sundström L, et al (2013) Transcriptome Characterisation of the ant *Formica exsecta* with new insights into the evolution of desaturase genes in social Hymenoptera. PLoS ONE 8: e68200. 23874539 10.1371/journal.pone.0068200 PMC3709892

[59] Wurm Y, Uva P, Ricci F, Wang J, Jemielity S, et al (2009) Fourmidable: a database for ant genomics. BMC Genomics 10: 5. 19126223 10.1186/1471-2164-10-5 PMC2639375

[60] Kurtz S, Phillippy A, Delcher AL, Smoot M, Shumway M, et al (2004) Versatile and open software for comparing large genomes. Genome Biol. 5: R12. 10.1186/gb-2004-5-2-r12 PMC395750 14759262

[61] Keller L, Liautard C, Reuter M, Brown WD, Sundström L, et al (2001) Sex ratio and *Wolbachia* infection in the ant *Formica exsecta* . Heredity 87: 227–233. 11703514 10.1046/j.1365-2540.2001.00918.x

[62] Duron O, Bouchon D, Boutin S, Bellamy L, Zhou L, et al (2008) The diversity of reproductive parasites among arthropods: *Wolbachia* do not walk alone. BMC Biol. 6: 27. 10.1186/1741-7007-6-27 PMC2492848 18577218

[63] Stevens L, Giordano R, Fialho RF (2001) Male-killing, nematode infections, bacteriophage infection, and virulence of cytoplasmic bacteria in the genus *Wolbachia* . Annu Rev Ecol Syst 32: 519–545.

[64] Russell JAR (2012) The ants (*Hymenoptera: Formicidae*) are unique and enigmatic hosts of prevalent *Wolbachia* (Alphaproteobacteria) symbionts, Myrmecol News. 16: 7–23.

[65] Troemel ER (2011) New models of microsporidiosis: infections in Zebrafish, *C. elegans*, and honey bee. PLoS Pathog. 7: e1001243. 10.1371/journal.ppat.1001243 PMC3040677 21379567

[66] Fuxa JR, Milks ML, Sokolova YY, Richter AR (2005) Interaction of an entomopathogen with an insect social form: an epizootic of *Thelohania solenopsae* (Microsporidia) in a population of the red imported fire ant, *Solenopsis invicta*. J. Invertebr. Pathol. 88: 79–82. 10.1016/j.jip.2004.10.001 15707872

[67] Knell JD, Allen GE, Hazard EI (1977) Light and electron microscope study of *Thelohania solenopsae* (Microsporida: Protozoa) in the red imported fire ant, *Solenopsis invicta*. J. Invertebr. Pathol. 29: 192–200. 10.1016/0022-2011(77)90193-8 850074

[68] Allen GE, Buren WF (2013) Microsporidan and fungal diseases of *Solenopsis invicta* Buren in Brazil. Journal of New York Entomological Society 82: 125–130.

[69] Jouvenaz DP, Ellis EA (1986) *Vairimorpha invictae* N. Sp. (Microspora: Microsporida), a parasite of the red imported fire ant, *Solenopsis invict*a Buren (Hymenoptera: Formicidae) 2. J. Eukaryot. Microbiol. 33: 457–461.

[70] Williams DF, Oi DH, Knue GJ (1999) Infection of red imported fire ant (*Hymenoptera: Formicidae*) colonies with the entomopathogen *Thelohania solenopsae* (Microsporidia: Thelohaniidae). J. Econ. Entomol. 92: 7. 10.1603/0022-0493-95.3.558 12076000

[71] Oi DH, Williams DF (2002) Impact of *Thelohania solenopsae* (Microsporidia: Thelohaniidae) on polygyne colonies of red imported fire ants (Hymenoptera: Formicidae). J. Econ. Entomol. 95: 558–562. 10.1603/0022-0493-95.3.558 12076000

[72] Briano JA, Williams DF (2002) Natural occurrence and laboratory studies of the fire ant pathogen *Vairimorpha invictae* (Microsporida: Burenellidae) in Argentina. Environ. Entomol. 31: 887–894.

[73] Oi DH, Briano JA, Valles SM, Williams DF (2005) Transmission of *Vairimorpha invictae* (Microsporidia: Burenellidae) infections between red imported fire ant (Hymenoptera: Formicidae) colonies. J. Invertebr. Pathol. 88: 108–115. 10.1016/j.jip.2004.11.006 15766927

[74] Darby AC, Choi J-H, Wilkes T, Hughes MA, Werren JH, et al (2010) Characteristics of the genome of *Arsenophonus nasoniae*, son-killer bacterium of the wasp *Nasonia*. Insect Mol. Biol. 19 Suppl 175–89. 10.1111/j.1365-2583.2009.00950.x 20167019

[75] Nováková E, Hypsa V, Moran NA (2009) *Arsenophonus*, an emerging clade of intracellular symbionts with a broad host distribution. BMC Microbiol. 9: 143. 10.1186/1471-2180-9-143 PMC2724383 19619300

[76] Funaro CF, Kronauer DJC, Moreau CS, Goldman-Huertas B, Pierce NE, et al (2011) Army ants harbor a host-specific clade of *Entomoplasmatales* bacteria. Appl. Environ. Microbiol. 77: 346–350. 10.1128/AEM.01896-10 PMC3019723 21075876

[77] Martinson VG, Danforth BN, Minckley RL, Rueppell O, Tingek S, et al (2011) A simple and distinctive microbiota associated with honey bees and bumble bees. Mol. Ecol. 20: 619–628. 10.1111/j.1365-294X.2010.04959.x 21175905

[78] Maori E, Lavi S, Mozes-Koch R, Gantman Y, Peretz Y, et al (2007) Isolation and characterization of Israeli acute paralysis virus, a dicistrovirus affecting honeybees in Israel: evidence for diversity due to intra- and inter-species recombination. J. Gen. Virol. 88: 3428–3438. 10.1099/vir.0.83284-0 18024913

[79] De MirandaJR, Drebot M, Tyler S, Shen M, Cameron CE, et al (2004) Complete nucleotide sequence of Kashmir bee virus and comparison with acute bee paralysis virus. J. Gen. Virol. 85: 2263–2270. 10.1099/vir.0.79990-0 15269367

[80] Valles SM, Strong CA, Dang PM, Hunter WB, Pereira RM, et al (2004) A picorna-like virus from the red imported fire ant, *Solenopsis invicta*: initial discovery, genome sequence, and characterization. Virology 328: 151–157. 15380366 10.1016/j.virol.2004.07.016

[81] Valles SM, Hashimoto Y (2009) Isolation and characterization of Solenopsis invicta virus 3, a new positive-strand RNA virus infecting the red imported fire ant, *Solenopsis invicta.* . Virology 388: 354–361. 19403154 10.1016/j.virol.2009.03.028

[82] Valles SM, Strong CA, Hashimoto Y (2007) A new positive-strand RNA virus with unique genome characteristics from the red imported fire ant, *Solenopsis invicta* . Virology 365: 457–463. 17477949 10.1016/j.virol.2007.03.043

[83] Valles SM, Oi DH, Yu F, Tan X-X, Buss EA (2012) Metatranscriptomics and pyrosequencing facilitate discovery of potential viral natural enemies of the invasive Caribbean crazy ant, *Nylanderia pubens* . PLoS ONE 7: e31828. 22384082 10.1371/journal.pone.0031828 PMC3288052

[84] Celle O, Blanchard P, Olivier V, Schurr F, Cougoule N, et al (2008) Detection of Chronic bee paralysis virus (CBPV) genome and its replicative RNA form in various hosts and possible ways of spread. Virus Res. 133: 280–284. 10.1016/j.virusres.2007.12.011 18243390

[85] Valles SM, Strong CA, Hunter WB, Dang PM, Pereira RM, et al (2008) Expressed sequence tags from the red imported fire ant, *Solenopsis invicta*: Annotation and utilization for discovery of viruses. J. Invertebr. Pathol. 99: 74–81. 10.1016/j.jip.2008.01.004 18329665

[86] Van Der Geest LPS, Elliot SL, Breeuwer JAJ, Beerling AAM (n.d.) Diseases of Mites. Exp. Appl. Acarol. 24: 497–560. 10.1023/a:1026518418163 11201358

[87] Cruickshank RH (2002) Molecular markers for the phylogenetics of mites and ticks. Syst Appl Acarol 7: 3–14.

[88] Sláviková E, Vadkertiová R (2003) The diversity of yeasts in the agricultural soil. J. Basic Microbiol. 43: 430–436. 10.1002/jobm.200310277 12964187

[89] Martini A, Applicata SM (1992) Biodiversity and conservation of yeasts. Biodivers. Conserv. 1 333: 324–333.

[90] Ba AS, Phillips SA (1996) Yeast biota of the red imported fire ant. Mycol. Res. 100: 740–746.

[91] Ribeiro MMR, Amaral KD, Seide VE, Souza BMR, Della Lucia TMC, et al (2012) Diversity of fungi associated with *Atta bisphaerica* (Hymenoptera: Formicidae): The activity of *Aspergillus ochraceus* and *Beauveria bassiana* . Psyche (Camb Mass) 2012: 1–6.

[92] Kidd SE, Sorrell TC, Meyer W (2003) Isolation of two molecular types of *Cryptococcus neoformans* var. *gattii* from insect frass. Med. Mycol. 41: 171–176. 10.1080/mmy.41.2.171.176 12964851

[93] Nilsson RH, Ryberg M, Kristiansson E, Abarenkov K, Larsson K-H, et al (2006) Taxonomic reliability of DNA sequences in public sequence databases: a fungal perspective. PLoS ONE 1: e59. 17183689 10.1371/journal.pone.0000059 PMC1762357

[94] Vänninen I (1996) Distribution and occurrence of four entomopathogenic fungi in Finland: effect of geographical location, habitat type and soil type. Mycol. Res. 100: 93–101.

[95] Reber A, Chapuisat M (2011) Diversity, prevalence and virulence of fungal entomopathogens in colonies of the ant *Formica selysi* . Insectes Soc 59: 231–239.

