# Correction: A Metatranscriptomic Approach to the Identification of Microbiota Associated with the Ant *Formica exsecta*

**DOI:** 10.1371/journal.pone.0121606

**Published:** 2015-03-13

**Authors:** The PLOS ONE Staff

In Table 1, there is an error in the title. Additionally, there are errors in the numbers of the FIMM and BGI columns of the New Stage in Caste/sex Queen. Please view a corrected version of Table 1 here.

**Table 1:** Number of individuals pooled in each of the total of 14 libraries sequenced by FIMM or BGI.

Caste/sex	Stage	FIMM	BGI
**Queen**	Old overwintered	6 (6)	4 (4)
	New	10 (5)	8 (4)
	Old cocoon	8 (4)	6 (3)
	Intermediate cocoon	8 (4)	6 (3)
	Young cocoon	8 (4)	6 (3)
**Worker**	Old overwintered	18 (6)	30 (8)
	New	10 (5)	15 (6)
	Old cocoon	8 (4)	6 (3)
	Intermediate cocoon	8 (4)	6 (3)
	Young cocoon	8 (4)	6 (3)
**Male**	Adult	3 (3)	3 (3)
	Old cocoon	3 (3)	3 (3)
	Intermediate cocoon	3 (3)	3 (3)
	Young cocoon	3 (3)	3 (3)

Castes and developmental classes of *F*. *exsecta* are given together with number of source colonies in parentheses.
