# Supplementary material for: A Metatranscriptomic Approach to the Identification of Microbiota Associated with the Ant Formica exsecta
Source: PLoS One. 2013 Nov 18;8(11):e79777. doi: 10.1371/journal.pone.0079777 (PMC3832538; doi:10.1371/journal.pone.0079777)
Supplement: Table S1 — Geographic localities, numbers of F. exsecta nests sampled and number of individuals from those nests. (DOCX) [file pone.0079777.s001.docx]

**Table S1. Geographic localities, numbers of *F. exsecta* nests sampled and number of individuals from those nests.**

| **habitat** | **locality** | **latitude** | **longitude** | **no. nests** | **no. individuals** |
| --- | --- | --- | --- | --- | --- |
| **islands** | Rovholmarna | 59.83738289 | 23.25233344 | 9 | 22 |
|  | Joskär | 59.84528216 | 23.25573171 | 14 | 33 |
|  | Furuskär | 59.83335445 | 23.27211607 | 17 | 57 |
| **mainland** | Harparskog | 59.93904411 | 23.33139143 | 2 | 10 |
|  | Prästkulla | 59.98031797 | 23.34493548 | 13 | 64 |
|  | Ingå | 60.06568368 | 23.85145684 | 3 | 23 |
|  |  |  | **Total:** | **58** | **209** |
