# Supplementary material for: A Metatranscriptomic Approach to the Identification of Microbiota Associated with the Ant Formica exsecta
Source: PLoS One. 2013 Nov 18;8(11):e79777. doi: 10.1371/journal.pone.0079777 (PMC3832538; doi:10.1371/journal.pone.0079777)
Supplement: Table S2 — Expression values by sex and caste for all sequence matches in Tables 2 – 4 , from both sequencing providers (Beijing Institute of Genomics, China, (BGI) and Finnish Institute of Molecular Medicine, Finland (FIMM). (DOCX) [file pone.0079777.s002.docx]

**Table S2. Expression values by sex and caste for all sequence matches in Tables 2-4, from both sequencing providers (Beijing Institute of Genomics, China, (BGI) and Finnish Institute of Molecular Medicine, Finland (FIMM). Contig names and fragment lengths (in basepairs) are given. Male = male cocoons and new male adults, NQ = new queen adults, OQ = old established colony queens, QC= queen cocoons, NW = new worker adults, OW = old (overwintered) worker adults, WC = worker cocoons.**

|  |  |  | **BGI** |  |  |  |  |  |  | **FIMM** |  |  |  |  |  |  |
| --- | --- | --- | --- | --- | --- | --- | --- | --- | --- | --- | --- | --- | --- | --- | --- | --- |
| **Organism (GenBank match)** | **Contig** | **length (bp)** | **Male** | **NQ** | **OQ** | **QC** | **NW** | **OW** | **WC** | **Male** | **NQ** | **OQ** | **QC** | **NW** | **OW** | **WC** |
| **Viruses, bacteria, fungi, mites (Table 2)** |  |  |  |  |  |  |  |  |  |  |  |  |  |  |  |  |
| Dicistrovirus | Contig22460 | 9554 | 0.90 | 396.08 | 0.00 | 6.09 | 3023.72 | 0.01 | 1.11 | 199.97 | 209.69 | 0.17 | 0.15 | 1106.99 | 0.15 | 0.22 |
| Iflavirus | Contig23147 | 9160 | 0.31 | 0.00 | 4.73 | 0.00 | 0.00 | 658.40 | 0.00 | 0.11 | 2316.77 | 0.16 | 0.13 | 3.45 | 0.17 | 0.19 |
| *Wolbachia_978* | Contig16923 | 2999 | 37.23 | 28.05 | 36.64 | 13.27 | 22.87 | 20.79 | 5.75 | 21.42 | 23.26 | 36.27 | 26.68 | 15.59 | 21.59 | 11.92 |
| *Wolbachia_416* | W_comp305_c0_seq1 | 1603 | 338.90 | 194.10 | 15.85 | 391.86 | 412.68 | 81.03 | 651.33 | 407.49 | 133.95 | 91.82 | 204.03 | 128.22 | 76.20 | 382.83 |
| *Encephalitozoon* | Contig41472 | 757 | 0.00 | 9.52 | 0.00 | 0.00 | 6.88 | 0.00 | 0.00 | 0.00 | 0.00 | 0.00 | 0.00 | 1.50 | 0.00 | 0.00 |
| *Cryptococcus* | M_comp34021_c0_seq1 | 456 | 5.21 | 0.30 | 0.14 | 0.82 | 0.19 | 0.49 | 1.97 | 0.00 | 0.00 | 0.00 | 0.00 | 0.16 | 0.15 | 0.35 |
| *Acidiphilium* | Contig28042 | 728 | 2953.18 | 2.31 | 5.76 | 2251.39 | 40.67 | 2.25 | 1920.64 | 100.48 | 1.21 | 1.47 | 460.57 | 0.75 | 0.92 | 405.51 |
| *Rhodococcus* | Contig39781 | 3238 | 753.59 | 655.48 | 878.21 | 598.22 | 1129.55 | 526.88 | 724.04 | 408.63 | 241.05 | 498.84 | 384.16 | 326.51 | 427.57 | 396.65 |
| *Aspergillus* | W_comp24854_c0_seq2 | 203 | 1.21 | 5.84 | 0.64 | 1.14 | 7.91 | 0.82 | 6.57 | 4.85 | 3.10 | 0.13 | 0.08 | 4.69 | 0.69 | 4.24 |
| *Penicillium* | Contig19642 | 282 | 4.22 | 11.20 | 27.56 | 37.00 | 12.82 | 1.00 | 14.28 | 0.32 | 0.33 | 0.00 | 0.17 | 0.97 | 0.49 | 0.35 |
| *Neurospora* | Q_comp234_c1_seq2 | 6803 | 482.20 | 285.86 | 41.86 | 325.84 | 371.59 | 282.02 | 341.26 | 42.18 | 91.43 | 13.08 | 185.41 | 184.37 | 72.65 | 168.87 |
| *Gibberella* | Contig17966 | 598 | 0.00 | 5.55 | 0.00 | 0.13 | 4.76 | 0.00 | 0.00 | 0.00 | 0.00 | 0.00 | 0.00 | 0.70 | 0.00 | 0.00 |
| *Podospora* | Contig4700 | 759 | 0.00 | 2.91 | 0.00 | 0.02 | 2.39 | 0.00 | 0.00 | 0.00 | 0.00 | 0.00 | 0.00 | 0.43 | 0.00 | 0.00 |
| *Eremothecium* | Q_comp26574_c0_seq3 | 324 | 0.28 | 9.18 | 0.00 | 0.47 | 4.06 | 0.06 | 5.43 | 0.35 | 0.39 | 0.00 | 0.00 | 5.59 | 0.14 | 0.37 |
| *Yarrowia* | Contig11501 | 1023 | 0.00 | 14.47 | 0.00 | 0.05 | 9.87 | 0.00 | 0.02 | 0.00 | 0.00 | 0.00 | 0.00 | 3.61 | 0.00 | 0.00 |
| *Ustilago* | Q_comp108872_c0_seq1 | 643 | 0.00 | 5.01 | 0.00 | 0.00 | 0.02 | 0.00 | 0.00 | 0.00 | 0.00 | 0.00 | 0.00 | 0.05 | 0.00 | 0.00 |
| *Varroa* | Contig13774, Q_comp104046_c0_seq1, Q_comp104046_c0_seq1, Q_comp10646_c0_seq1, Q_comp11073_c0_seq1, Q_comp22427_c0_seq1, Q_comp29293_c0_seq1, Q_comp91137_c0_seq1 | - | 1.15 | 27.27 | 0.04 | 1.28 | 2.07 | 0.21 | 1.62 | 8.31 | 3.91 | 4.54 | 2.78 | 4.90 | 1.80 | 4.31 |
| *Candida* | Q_comp204272_c0_seq1 | 200 | 0.00 | 2.47 | 0.00 | 0.13 | 0.55 | 0.00 | 0.00 | 0.00 | 0.00 | 0.00 | 0.00 | 0.00 | 0.00 | 0.00 |
| *Saccharomyces* | Q_comp216446_c0_seq1 | 213 | 0.00 | 2.49 | 0.00 | 0.00 | 0.11 | 0.00 | 0.00 | 0.00 | 0.00 | 0.00 | 0.00 | 0.00 | 0.00 | 0.00 |
| *Scizosaccharomyces* | Q_comp119310_c0_seq1 | 248 | 0.00 | 3.73 | 0.00 | 0.16 | 0.15 | 0.00 | 0.00 | 0.00 | 0.00 | 0.00 | 0.00 | 0.00 | 0.00 | 0.00 |
| *Zygosaccaromyces* | Q_comp169804_c0_seq1 | 271 | 0.00 | 2.82 | 0.00 | 0.09 | 0.85 | 0.00 | 0.00 | 0.00 | 0.00 | 0.00 | 0.00 | 0.81 | 0.00 | 0.00 |
| *Kluyveromyces* | W_comp19498_c0_seq1 | 185 | 1.72 | 9.07 | 0.25 | 2.29 | 10.39 | 0.16 | 23.99 | 1.93 | 0.68 | 0.22 | 0.71 | 4.16 | 0.75 | 0.32 |
| *Naumovozyma* | Q_comp136067_c0_seq1 | 196 | 0.09 | 4.03 | 0.00 | 0.13 | 0.31 | 0.00 | 0.00 | 0.00 | 0.00 | 0.00 | 0.00 | 0.09 | 0.00 | 0.00 |
| *Tetrapisispora* | Contig1577 | 2032 | 267.00 | 439.43 | 112.68 | 156.68 | 179.63 | 126.78 | 91.29 | 121.74 | 366.00 | 165.54 | 297.04 | 132.93 | 174.00 | 150.48 |
| *Torulaspora* | Contig15436 | 232 | 0.00 | 12.40 | 0.00 | 0.11 | 19.72 | 0.00 | 0.00 | 0.00 | 0.27 | 0.00 | 0.00 | 16.18 | 0.00 | 0.00 |
| **18S rRNA mites (Table 3)** |  |  |  |  |  |  |  |  |  |  |  |  |  |  |  |  |
| *Acarus* | comp153878_c0_seq1 | 113 | 0.00 | 1.20 | 0.00 | 0.11 | 1.40 | 0.00 | 0.00 | 0.23 | 0.00 | 0.00 | 0.00 | 0.49 | 0.00 | 0.00 |
| *Ewingia* | comp119450_c0_seq1 | 111 | 0.00 | 0.78 | 0.00 | 0.00 | 3.62 | 0.00 | 0.00 | 0.12 | 0.28 | 0.00 | 0.00 | 2.47 | 0.42 | 0.00 |
| *Histiostoma* | comp35793_c0_seq1, comp37322_c0_seq1, comp53068_c0_seq1 | - | 0.98 | 4.97 | 0.00 | 0.24 | 44.24 | 0.00 | 0.00 | 0.12 | 0.48 | 0.00 | 0.14 | 19.90 | 2.21 | 0.19 |
| *Tyroborus* | comp100494_c0_seq1 | 125 | 0.07 | 5.04 | 0.00 | 0.00 | 6.23 | 0.00 | 0.00 | 0.51 | 0.00 | 0.00 | 0.00 | 0.73 | 0.56 | 0.00 |
| *Holostaspis* | comp33306_c0_seq1, comp402454_c0_seq1 | - | 0.00 | 22.33 | 0.00 | 0.11 | 0.72 | 0.00 | 0.00 | 0.00 | 0.00 | 0.00 | 0.00 | 0.00 | 0.00 | 0.00 |
| *Gaeolaelaps* | comp32465_c0_seq1 | 155 | 0.00 | 3.26 | 0.00 | 0.00 | 0.00 | 0.00 | 0.00 | 0.00 | 0.00 | 0.00 | 0.00 | 0.00 | 0.00 | 0.00 |
| *Veigaia* | comp242201_c0_seq1 | 111 | 0.00 | 3.56 | 0.00 | 0.12 | 0.00 | 0.00 | 0.00 | 0.46 | 1.13 | 0.24 | 0.15 | 0.66 | 0.42 | 0.45 |
| **16S rRNA bacteria (Table 4)** |  |  |  |  |  |  |  |  |  |  |  |  |  |  |  |  |
| *Micrococcineae* | Q_comp50162_c0_seq1 | 286 | 0.89 | 2.42 | 0.10 | 7.62 | 15.74 | 0.00 | 5.39 | 0.22 | 0.88 | 0.56 | 0.17 | 3.14 | 0.97 | 0.38 |
| *Micrococcineae* | W_comp10272_c0_seq2 | 485 | 1.63 | 3.51 | 0.74 | 5.26 | 13.90 | 0.16 | 9.25 | 0.29 | 0.26 | 0.06 | 0.00 | 2.53 | 0.05 | 0.06 |
| *Micrococcineae* | W_comp29937_c0_seq1 | 274 | 0.76 | 2.39 | 0.07 | 7.58 | 16.08 | 0.00 | 5.53 | 0.14 | 0.57 | 0.39 | 0.18 | 2.94 | 0.85 | 0.40 |
| *Pedobacter* | Q_comp98495_c0_seq1 | 330 | 0.03 | 5.50 | 0.00 | 0.00 | 0.00 | 0.00 | 0.00 | 0.00 | 0.00 | 0.00 | 0.00 | 0.00 | 0.07 | 0.03 |
| *Streptococcus* | Q_comp184401_c0_seq1 | 224 | 0.16 | 1.32 | 0.00 | 1.37 | 0.76 | 0.00 | 0.54 | 0.00 | 0.00 | 0.12 | 0.00 | 0.00 | 0.10 | 0.00 |
| *Lactobacillus* | W_comp10272_c0_seq5 | 257 | 3.08 | 18.44 | 0.18 | 0.25 | 20.74 | 0.19 | 0.14 | 20.79 | 4.04 | 1.35 | 0.19 | 7.19 | 0.54 | 0.51 |
| *Saccharibacter* | Contig1467 | 261 | 7.60 | 10.69 | 0.50 | 0.05 | 24.48 | 1.01 | 0.05 | 7.44 | 5.55 | 0.72 | 0.00 | 8.35 | 0.98 | 0.12 |
| *Saccharibacter* | Contig77 | 279 | 6.32 | 22.07 | 0.83 | 0.37 | 26.74 | 0.35 | 0.00 | 17.77 | 8.57 | 2.15 | 0.00 | 12.66 | 1.25 | 0.14 |
| *Kozakia* | W_comp10272_c0_seq4 | 345 | 7.12 | 15.10 | 1.23 | 4.79 | 35.63 | 0.99 | 3.69 | 11.52 | 4.56 | 0.81 | 0.24 | 9.71 | 0.61 | 0.64 |
| *Anaplasma* | M_comp278_c0_seq1 | 1479 | 366.20 | 209.23 | 16.96 | 422.80 | 445.89 | 85.84 | 703.28 | 443.27 | 144.91 | 99.29 | 220.73 | 138.34 | 82.35 | 414.19 |
| *Burkholderia* | W_comp17436_c0_seq7 | 239 | 0.04 | 0.15 | 0.04 | 0.05 | 6.47 | 0.00 | 0.00 | 0.00 | 0.40 | 0.00 | 0.00 | 0.84 | 0.00 | 0.08 |
| *Burkholderia* | W_comp67223_c0_seq1 | 241 | 0.38 | 0.61 | 0.12 | 1.28 | 11.31 | 0.85 | 0.91 | 0.05 | 0.13 | 0.00 | 0.14 | 5.24 | 0.00 | 0.50 |
| *Arsenophonus* | W_comp107091_c0_seq1 | 265 | 0.00 | 0.00 | 0.38 | 0.00 | 0.00 | 3.79 | 0.00 | 0.05 | 0.00 | 0.05 | 0.19 | 0.00 | 0.00 | 0.11 |
| *Enhydrobacter* | Q_comp5639_c0_seq1 | 418 | 1.76 | 3.90 | 2.24 | 9.11 | 7.34 | 0.23 | 13.64 | 0.40 | 0.15 | 0.29 | 0.28 | 3.37 | 2.00 | 1.63 |
| *Acinetobacter* | Q_comp5639_c0_seq2 | 417 | 2.25 | 3.85 | 2.29 | 8.39 | 5.87 | 0.12 | 9.13 | 0.49 | 0.08 | 0.19 | 0.16 | 1.98 | 1.39 | 1.20 |
| *Azomonas* | Contig16461 | 414 | 3.73 | 6.44 | 16.00 | 20.06 | 11.17 | 1.18 | 11.92 | 0.93 | 0.15 | 0.03 | 0.08 | 3.10 | 0.62 | 2.97 |
| *Azomonas* | W_comp17436_c0_seq2 | 515 | 1.24 | 5.75 | 11.06 | 10.20 | 14.44 | 0.98 | 6.05 | 0.47 | 0.37 | 0.05 | 0.06 | 3.09 | 0.50 | 2.27 |
| *Pseudomonas* | Contig19642 | 282 | 4.22 | 11.20 | 27.56 | 37.00 | 12.82 | 1.00 | 14.28 | 0.32 | 0.33 | 0.00 | 0.17 | 0.97 | 0.49 | 0.35 |
| *Entomoplasma* | Contig20972 | 581 | 0.03 | 6.48 | 0.00 | 0.09 | 36.91 | 0.00 | 0.08 | 0.70 | 0.54 | 0.02 | 0.06 | 5.32 | 0.00 | 0.00 |
| *Entomoplasma* | Contig30367 | 261 | 0.00 | 7.61 | 0.00 | 0.05 | 28.63 | 0.00 | 0.00 | 0.00 | 0.00 | 0.00 | 0.00 | 4.49 | 0.00 | 0.00 |
| *Mesoplasma* | Contig30477 | 218 | 0.08 | 9.06 | 0.25 | 1.00 | 29.76 | 0.04 | 0.50 | 0.00 | 0.00 | 0.00 | 0.15 | 2.35 | 0.00 | 0.00 |
