# Supplementary material for: A Metatranscriptomic Approach to the Identification of Microbiota Associated with the Ant Formica exsecta
Source: PLoS One. 2013 Nov 18;8(11):e79777. doi: 10.1371/journal.pone.0079777 (PMC3832538; doi:10.1371/journal.pone.0079777)
Supplement: Table S3 — RDP classifier classification of the fungal large subunit (LSU) gene sequences. (DOCX) [file pone.0079777.s003.docx]

**Table S3. RDP classifier classification of the fungal large subunit (LSU) gene sequences. *Fungi i.s.= Fungi incertae sedis.***

| **Sequence name** | **Phylum** | **Confidence** | **Class** | **C** | **Order** | **C** | **Family** | **C** | **Genus** | **Confidence** |
| --- | --- | --- | --- | --- | --- | --- | --- | --- | --- | --- |
| comp64065_c0_seq1 | *Ascomycota* | 100% | *Lecanoromycetes* | 100% | *Lecanorales* | 100% | *Parmeliaceae* | 100% | *Parmotrema* | 100% |
| comp77624_c0_seq1 | *Ascomycota* | 100% | *Sordariomycetes* | 100% | *Phyllachorales* | 93% | *Phyllachoraceae* | 93% | *Plectosphaerella* | 93% |
| comp146017_c0_seq1 | *Ascomycota* | 100% | *Sordariomycetes* | 100% | *Phyllachorales* | 100% | *Phyllachoraceae* | 100% | *Plectosphaerella* | 100% |
| comp38364_c0_seq2 | *Ascomycota* | 37% | *Taphrinomycetes* | 19% | *Taphrinales* | 19% | *Protomycetaceae* | 19% | *Protomyces* | 19% |
| comp232879_c0_seq1 | *Ascomycota* | 45% | *Lecanoromycetes* | 6% | *Lecanorales* | 3% | *Physciaceae* | 2% | *Anaptychia* | 2% |
| comp17115_c0_seq1 | *Ascomycota* | 48% | *Lecanoromycetes* | 14% | *Lecanorales* | 8% | *Hymeneliaceae* | 3% | *Hymenelia* | 3% |
| comp75348_c0_seq1 | *Ascomycota* | 53% | *Lecanoromycetes* | 19% | *Acarosporales* | 12% | *Acarosporaceae* | 12% | *Glypholecia* | 12% |
| comp66134_c0_seq3 | *Ascomycota* | 53% | *Pezizomycetes* | 6% | *Pezizales* | 6% | *Pyronemataceae* | 6% | *Tricharina* | 4% |
| comp36505_c0_seq1 | *Ascomycota* | 54% | *Sordariomycetes* | 8% | *Coronophorales* | 5% | *Coronophorales i.s.* | 4% | *Spinulosphaeria* | 4% |
| comp76547_c0_seq1 | *Ascomycota* | 56% | *Lecanoromycetes* | 19% | *Lecanorales* | 9% | *Lecanoraceae* | 4% | *Lecidella* | 4% |
| comp99570_c0_seq1 | *Ascomycota* | 61% | *Sordariomycetes* | 22% | *Chaetosphaeriales* | 4% | *Chaetosphaeriaceae* | 4% | *Carpoligna* | 3% |
| comp17115_c0_seq4 | *Ascomycota* | 61% | *Sordariomycetes* | 22% | *Sordariales* | 9% | *Chaetomiaceae* | 9% | *Zopfiella* | 9% |
| comp115328_c0_seq1 | *Ascomycota* | 61% | *Dothideomycetes* | 10% | *Pleosporales* | 8% | *Pleomassariaceae* | 1% | *Pleomassaria* | 1% |
| comp17115_c0_seq2 | *Ascomycota* | 62% | *Lecanoromycetes* | 16% | *Ostropales* | 9% | *Stictidaceae* | 8% | *Acarosporina* | 8% |
| comp185304_c0_seq1 | *Ascomycota* | 65% | *Leotiomycetes* | 18% | *Helotiales* | 18% | *Hemiphacidiaceae* | 13% | *Fabrella* | 13% |
| comp70529_c0_seq2 | *Ascomycota* | 67% | *Sordariomycetes* | 25% | *Sordariomycetes i.s.* | 20% | *Sordariomycetes i.s.* | 20% | *Torpedospora* | 18% |
| comp120517_c0_seq1 | *Ascomycota* | 68% | *Sordariomycetes* | 26% | *Sordariales* | 2% | *Chaetomiaceae* | 0% | *Zopfiella* | 0% |
| comp124963_c0_seq1 | *Ascomycota* | 70% | *Lecanoromycetes* | 36% | *Lecanorales* | 26% | *Psoraceae* | 11% | *Lecidoma* | 11% |
| comp303848_c0_seq1 | *Ascomycota* | 72% | *Lichinomycetes* | 5% | *Lichinales* | 5% | *Lichinaceae* | 5% | *Lempholemma* | 5% |
| comp15208_c0_seq1 | *Ascomycota* | 87% | *Lecanoromycetes* | 59% | *Lecanorales* | 57% | *Parmeliaceae* | 56% | *Parmotrema* | 56% |
| comp101818_c0_seq1 | *Ascomycota* | 91% | *Saccharomycetes* | 61% | *Saccharomycetales* | 61% | *Trichomonascaceae* | 24% | *Wickerhamiella* | 24% |
| comp105995_c0_seq1 | *Ascomycota* | 96% | *Sordariomycetes* | 82% | *Hypocreales* | 24% | *Niessliaceae* | 17% | *Emericellopsis* | 17% |
| comp30322_c0_seq1 | *Ascomycota* | 99% | *Lecanoromycetes* | 99% | *Lecanorales* | 99% | *Parmeliaceae* | 99% | *Parmotrema* | 99% |
| comp23800_c0_seq2 | *Basidiomycota* | 100% | *Agaricomycetes* | 100% | *Agaricales* | 100% | *Pleurotaceae* | 100% | *Hohenbuehelia* | 100% |
| comp105847_c0_seq3 | *Basidiomycota* | 100% | *Agaricomycetes* | 100% | *Agaricales* | 100% | *Pleurotaceae* | 100% | *Hohenbuehelia* | 100% |
| comp70529_c0_seq1 | *Basidiomycota* | 27% | *Tremellomycetes* | 10% | *Tremellales* | 10% | *Sirobasidiaceae* | 10% | *Fibulobasidium* | 10% |
| comp31504_c0_seq1 | *Basidiomycota* | 32% | *Agaricomycetes* | 27% | *Agaricales* | 25% | *Pleurotaceae* | 23% | *Hohenbuehelia* | 23% |
| comp247403_c0_seq1 | *Basidiomycota* | 63% | *Microbotryomycetes* | 31% | *Microbotryales* | 19% | *Ustilentylomataceae* | 19% | *Ustilentyloma* | 11% |
| comp52835_c0_seq1 | *Basidiomycota* | 98% | *Agaricomycetes* | 97% | *Agaricales* | 97% | *Pleurotaceae* | 97% | *Hohenbuehelia* | 97% |
| comp105847_c0_seq2 | *Basidiomycota* | 98% | *Agaricomycetes* | 98% | *Agaricales* | 98% | *Pleurotaceae* | 98% | *Hohenbuehelia* | 98% |
| comp44352_c0_seq2 | *Blastocladiomycota* | 12% | *Blastocladiomycetes* | 12% | *Blastocladiales* | 12% | *Coelomomycetaceae* | 12% | *Coelomomyces* | 12% |
| comp66134_c0_seq1 | *Blastocladiomycota* | 12% | *Blastocladiomycetes* | 12% | *Blastocladiales* | 12% | *Coelomomycetaceae* | 12% | *Coelomomyces* | 12% |
| comp17115_c0_seq3 | *Blastocladiomycota* | 12% | *Blastocladiomycetes* | 12% | *Blastocladiales* | 12% | *Blastocladiaceae* | 10% | *Blastocladiella* | 7% |
| comp432674_c0_seq1 | *Blastocladiomycota* | 16% | *Blastocladiomycetes* | 16% | *Blastocladiales* | 16% | *Catenariaceae* | 14% | *Catenomyces* | 12% |
| comp230755_c0_seq1 | *Blastocladiomycota* | 21% | *Blastocladiomycetes* | 21% | *Blastocladiales* | 21% | *Catenariaceae* | 15% | *Catenomyces* | 14% |
| comp90910_c0_seq2 | *Blastocladiomycota* | 21% | *Blastocladiomycetes* | 21% | *Blastocladiales* | 21% | *Coelomomycetaceae* | 17% | *Coelomomyces* | 17% |
| comp90910_c0_seq1 | *Blastocladiomycota* | 21% | *Blastocladiomycetes* | 21% | *Blastocladiales* | 21% | *Coelomomycetaceae* | 17% | *Coelomomyces* | 17% |
| comp46218_c0_seq1 | *Blastocladiomycota* | 60% | *Blastocladiomycetes* | 60% | *Blastocladiales* | 60% | *Blastocladiaceae* | 24% | *Microallomyces* | 13% |
| comp75348_c0_seq4 | *Chytridiomycota* | 19% | *Chytridiomycetes* | 19% | *Chytridiales* | 16% | *Chytridiales i.s.* | 16% | *Batrachochytrium* | 16% |
| comp36055_c0_seq1 | *Chytridiomycota* | 8% | *Chytridiomycetes* | 8% | *Spizellomycetales* | 5% | *Spizellomycetaceae* | 4% | *Spizellomyces* | 4% |
| comp44766_c0_seq1 | *Fungi i.s.* | 12% | *Fungi i.s.* | 12% | *Zoopagales* | 12% | *Piptocephalidaceae* | 10% | *Kuzuhaea* | 5% |
| comp93519_c0_seq1 | *Fungi i.s.* | 13% | *Fungi i.s.* | 12% | *Zoopagales* | 12% | *Helicocephalidaceae* | 7% | *Rhopalomyces* | 7% |
| comp42140_c0_seq1 | *Fungi i.s.* | 15% | *Ichthyosporea* | 11% | *Ichthyophonida* | 11% | *Amoebidiaceae* | 11% | *Amoebidium* | 11% |
| comp25384_c0_seq1 | *Fungi i.s.* | 22% | *Fungi i.s.* | 22% | *Zoopagales* | 22% | *Helicocephalidaceae* | 20% | *Rhopalomyces* | 20% |
| comp62633_c0_seq1 | *Neocallimastigomycota* | 22% | *Neocallimastigomycetes* | 22% | *Neocallimastigales* | 22% | *Neocallimastigaceae* | 22% | *Cyllamyces* | 8% |
| comp230755_c0_seq2 | *Neocallimastigomycota* | 24% | *Neocallimastigomycetes* | 24% | *Neocallimastigales* | 24% | *Neocallimastigaceae* | 24% | *Cyllamyces* | 13% |
